# Synthesis and evaluation of substituted diphenyl-1,3,4-oxadiazole derivatives for central nervous system depressant activity

**DOI:** 10.1186/2191-2858-2-8

**Published:** 2012-03-01

**Authors:** Poonam Singh, Pramod Kumar Sharma, Jitendra Kumar Sharma, Anshu Upadhyay, Nitin Kumar

**Affiliations:** 1 Department of Pharmaceutical Technology, Meerut Institute of Engineering & Technology, NH-58, Baghpat By-pass Crossing, Delhi-Haridwar Highway, Meerut-250005, India; 2 Faculty of Pharmacy, Dehradun Institute of Technology, Dehradun, Uttarakhand, India

## Abstract

**Background:**

Substituted 1,3,4-oxadiazoles are of considerable pharmaceutical interest. 2,5-Substituted diphenyl-1,3,4-oxadiazoles are associated with diverse biological activities by the virtue of -N = C-O- grouping. In the view of wide range of biological properties associated with 1,3,4-oxadiazole, we have synthesized substituted derivatives of 1,3,4-oxadiazole (**XIII-XXII**), a versatile hydrophobic molecule possessing preliminary CNS properties, with the hope to potentiate the biological activities with lesser or limited amount of toxicities.

**Method:**

The synthesis was based on ester substitution of substituted benzohydrazide in presence of hydrazine hydrate followed by cyclization in presence of phosphorus oxychloride. All the synthesized compounds were evaluated for their potential CNS depressant activities. Statistical analysis of the anticonvulsant, antidepressant, and antianxiety activity of the synthesized compounds on animals was evaluated using one-way analysis of variance (ANOVA).

**Results:**

Two compounds 5-(4-nitrophenyl)-2-(4-chlorophenyl)-1,3,4-oxadiazole (**XIV**) and 5-(4-nitrophenyl)-2-(4-nitrophenyl)-1,3,4-oxadiazole (**XV**) were found to be the most promising compounds of the series in antidepressant, anticonvulsant and antianxiety activity with no neurotoxicity when compared with standard.

**Conclusions:**

Among the synthesized compounds, it was found that incorporation of electron withdrawing group at C2 and C5 position of the oxadiazole ring led to high degree of pharmacological activity. Thus compounds 5-(4-nitrophenyl)-2-(4-chlorophenyl)-1,3,4-oxadiazole **(XIV) **and 5-(4-nitrophenyl)-2-(4-nitrophenyl)-1,3,4-oxadiazole (**XV) **showed excellent CNS depressant activities. The result of the present investigation may encourage us to develop and/or improve similar other related compounds and it may be assumed that further modifications may produce compounds of better activity with lesser side effects.

## Background

Epilepsy is one of the most common neurological disorders responsible for substantial morbidity and mortality. It is a chronic neurological disorder characterized by paroxysmal, excessive, and hyper-synchronous neuronal activity in the brain affecting around 1-2% of the world population [1,2]. 75-80% of the epileptic patients may be provided with adequate seizure control with the help of conventional antiepileptic drugs. Despite the development of several new anticonvulsants, the treatments of epilepsy still remain inadequate. However, over 30% of people with epilepsy do not have seizure control even with the best available medications [3].

Most people with epilepsy have a normal emotional and cognitive life, however neurobehavioral problems can be found in a large number of patients. Higher rates of psychopathology have been reported in people with epilepsy compared with the general population and in people with chronic non-neurological disorders [4]. Depression and anxiety are the most frequent types of psychiatric disorders identified in patients with epilepsy [5]. There are numerous studies reporting that the severity of anxiety and depression in epileptic patients is higher than normal controls [6-8].

The comorbidity of major depressive disorder or anxiety is associated with medical, psychiatric, and social problems. A bidirectional relationship between epilepsy and depression is supposed to exist and studies suggested that depression and epilepsy may share common pathogenic mechanisms and the occurrence of one may facilitate the development of the other and vice versa [9,10]. Multiple factors are implicated in the development of depression in epilepsy including clinical (seizure frequency, seizure type or foci, epilepsy duration, age at onset, antiepileptic drugs) and psychosocial ones (life stressors, employment, marital status, quality of life) [11]. Here we have selected an *in-vivo *efficacy approach using maximal electroshock seizure (MES) model, spontaneous motor activity using actophotometer and elevated plus maze model. Benzodiazepines (BZDs) are the most frequently prescribed synthetic drugs for variety of conditions particularly anxiety, depression, epilepsy, and insomnia. But these psychoneural drugs have many side effects including addictive potential and frequent development of tolerance. BZDs have limited utility for long-term treatment. Therefore our strategy was to find drugs which are effective in all these models and are better tolerated than the BZDs and other conventional drugs.

## Results and discussion

### Chemistry

Although various methods have been reported for the synthesis of oxadiazole derivatives [12] but these methods requires high reaction temperature, long reaction, time and expensive reagents. In present described experimental conditions the heterocyclization reaction reached completion with a relative simple operation giving high yield (65-95%) at shorter time (1.5-3.0 h) compared to the other reported cyclization methods for 1,3,4-oxadiazole ring. The synthetic route of the designed 1,3,4-oxadiazole derivatives 2,5-substituted diphenyl-1,3,4-oxadiazole (**XIII-XXII**) was outlined in Scheme 1. The possible mechanism of the final step involved in formation of target compounds was shown in Scheme 2. The presence of electron donating groups (-NH2, -OH) decreases the yield of the final compound (50-75%) with low reaction time (1.5-2.0 h) whereas the presence of electron withdrawing groups (-NO2, -Cl, -Br) increases the yield of the final compound thereby increasing the reaction time (2.5-3.0 h). The structures of the compounds were assigned on the basis of elemental analysis, IR, ^1^H NMR, and mass spectra. The structures of newly synthesized compounds were confirmed by spectral and analytical data.

In general, the IR spectra of synthesized compounds showed a C = N stretching band around 1533-1684 cm^-1^, C = O absorption band at around 1010-1091 cm^-1^, and C-O-C absorption band at around 1239-1282 cm^-1 ^indicating formation of the 1,3,4-oxadiazole ring. In ^1^H NMR spectra, the signals of respective protons of newly synthesized compounds showed the peaks for -NH, -OH, and Ar-H near δ 3.8-4.9, 4.3-5.6, and 6.52-8.61, respectively. Further evidence for the formation of 2,5-substituted diphenyl-1,3,4-oxadiazole was obtained by recording the mass spectra. The general mass fragmentation pattern for the compound XIII showed the molecular ion peak at 272.69 which is in conformity with the molecular formula C14H9ClN2O2 (Scheme 3). Both analytical and spectral data (IR, ^1^H NMR, and mass) of all the synthesized compounds were in full agreement with the proposed structures.

### Pharmacology

**Figure 1:**
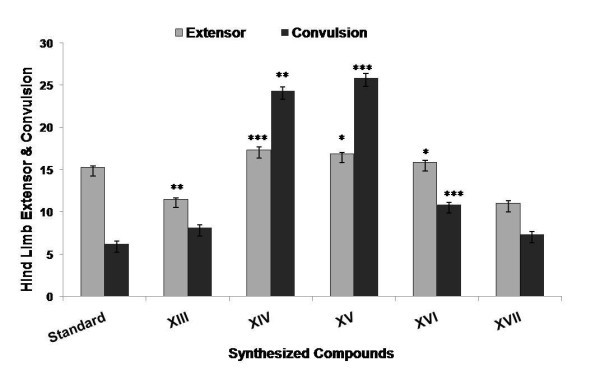
**Anticonvulsant activity of synthesized compounds at the dose (30 mg kg^-1^, p.o.) in female albino mice for single administration**.

**Figure 2:**
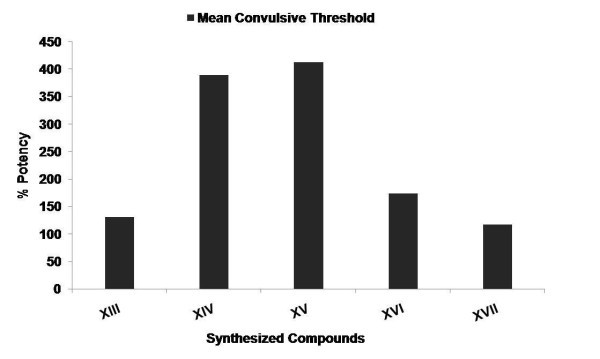
**%Potency of synthesized compounds at the dose (30 mg kg^-1^, p.o.) in female albino mice for single administration**. All the values were expressed as mean convulsive threshold.

**Figure 3:**
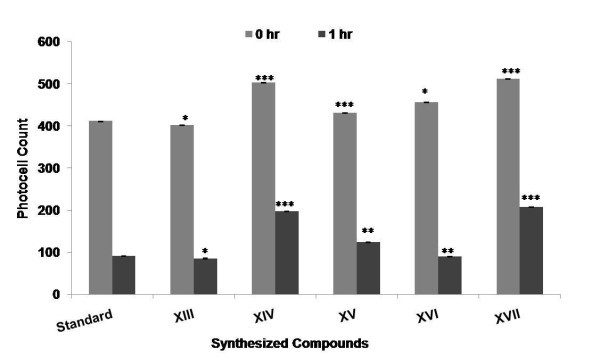
**Antidepressant activity of synthesized compounds at the dose (10 mg kg^-1^, p.o.) showing number of photocell count**.

**Figure 4:**
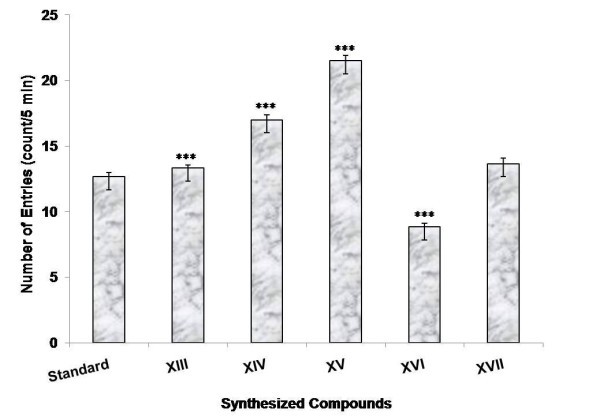
**Antianxiety activity of synthesized compounds at the dose (2 mg kg^-1^, p.o.) showing number of entries 5 min^-1^**.

**Figure 5:**
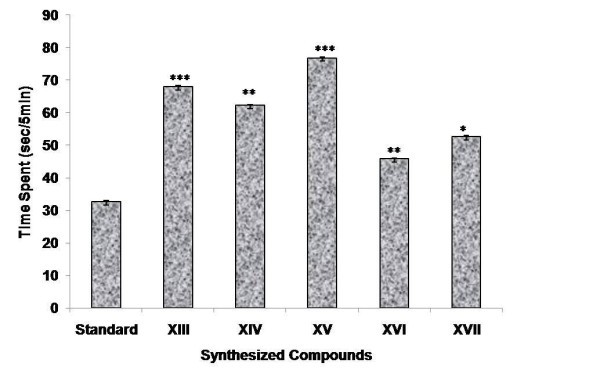
**Antianxiety activity of synthesized compounds at the dose (2 mg kg^-1^, p.o.) showing time spent by mouse in open arm**.

Among the compounds synthesized (**XIII-XXII**), few compounds were selected (**XIII-XVII**) and evaluated for CNS depressant activities such as antidepressant, anticonvulsant, antianxiety, and neurotoxicity activity. The pharmacological data indicated that among all the compounds being screened, compounds **XIV **and **XV **showed highly significant anticonvulsant activity (***p ≤ 0.001) and compound **XVI **showed less significant activity when compared to the standard (Table 1 and Figure 1). The % potency showing the mean convulsive threshold of the synthesized compounds was shown in Figure 2. In antidepressant screening, compounds **XIV**, **XV**, and **XVII **decreased the locomotor activity significantly (***p ≤ 0.001) whereas, compounds **XVI **(**p ≤ 0.01) and **XIII **(*p ≤ 0.05) did not showed a significant reduction in the locomotor activity when compared to standard fluoxetine (Table 2 and Figure 3). In antianxiety screening, the number of entries and the mean time spent in open arm has been shown in (Table 3, Figures 4 and 5) and compounds, **XIII **and **XV **significantly increases the time spent in open arm thus showing higher activity when compared to the standard diazepam. Whereas another remarkable point is that compound having nitro group (electron withdrawing groups) at both position 2 and 5 of the 1,3,4-oxadiazole ring, i.e., 5-(4-nitrophenyl)-2-(4-nitrophenyl)-1,3,4-oxadiazole **(XV) **exhibited greater anticonvulsant, antidepressant, and antianxiety activity as compared to the other compounds of the series. Whereas compound 5-(4-nitrophenyl)-2-(4-chlorophenyl)-1,3,4-oxadiazole (**XIV**) showed highly significant anticonvulsant and antidepressant activity but less significant antianxiety activity. In the neurotoxicity screen, all the compounds being screened passed the neurotoxicity testing, i.e., not showing neurological deficit (Table 4). From the overall result it is evident that compound **XIV **and **XV **could be identified as the most biologically active member within this study with good anticonvulsant, antidepressant, and antianxiety activity.

**Table 1:** Anticonvulsant activity of synthesized compounds

Number	Compound	Dose (mg kg^-1^)	Hind limb extensor (mean ± S.E.M)	Hind limb convulsion (mean ± S.E.M)	% Potency
1.	Control	30	27.50 ± 0.42	6.5 ± 0.47	-
2.	Standard	30	15.25 ± 0.17	6.25 ± 0.30	-
3.	XIII	30	11.50 ± 0.15**	8.16 ± 0.30	130.672
4.	XIV	30	17.33 ± 0.33***	24.33 ± 0.42**	389.28
5.	XV	30	16.83 ± 0.20*	25.83 ± 0.54***	413.28
6.	XVI	30	15.83 ± 0.27*	10.83 ± 0.30***	173.28
7.	XVII	30	11.00 ± 0.30	7.33 ± 0.33	117.32

Values are mean ± SEM of six animals in each group. Statistical significance versus standard (***p ≤ 0.001, **p ≤ 0.01, *p ≤ 0.05)

**Table 2:** Antidepressant activity of synthesized compounds

Number	Compound	Dose (mg kg^-1^)	0 h (mean ± S.E.M)	1 h (mean ± S.E.M)
1.	Control	10	345.83 ± 0.27	303.21 ± 0.42
2.	Standard	10	411.16 ± 0.30	91.33 ± 0.21
3.	XIII	10	401.50 ± 0.22*	85.16 ± 0.47*
4.	XIV	10	502.83 ± 0.47 ***	196.66 ± 0.42***
5.	XV	10	431.16 ± 0.30***	124.16 ± 0.40**
6.	XVI	10	455.83 ± 0.47*	89.16 ± 0.30**
7.	XVII	10	511.50 ± 0.42***	207.00 ± 0.36***

Values are mean ± SEM of six animals in each group. Statistical significance versus standard (***p ≤ 0.001, **p ≤ 0.01, *p ≤ 0.05)

**Table 3:** Antianxiety activity of synthesized compounds

Number	Compound	Dose (mg kg^-1^)	Number of entries in open arms (Mean ± S.E.M)	Average time spent in open arms (Mean ± S.E.M)
1.	Control	10	2.10 ± 0.23	9.77 ± 0.27
2.	Standard	10	12.66 ± 0.33	32.66 ± 0.33
3.	XIII	10	13.33 ± 0.21***	68.00 ± 0.36***
4.	XIV	10	17.00 ± 0.36***	62.33 ± 0.21**
5.	XV	10	21.50 ± 0.42***	76.83 ± 0.30***
6.	XVI	10	8.83 ± 0.30***	45.83 ± 0.30**
7.	XVII	10	13.66 ± 0.42	52.66 ± 0.33*

Values are mean ± SEM of six animals in each group. Statistical significance versus standard (***p ≤ 0.001, **p ≤ 0.01, *p ≤ 0.05)

**Table 4:** Neurotoxic activity of the synthesized compounds

Compound	Dose (mg kg^-1^)	n	Result
Std. (Phenytoin)	30	6	X
XIII	30	6	-
XIV	30	6	X
XV	30	6	X
XVI	30	6	-
XVIII	30	6	X

*n*, number of mice = 6; X, does not show neurotoxicity; ROA, orally; -, neurotoxicity not checked.

## Conclusions

This preliminary investigation showed the cyclization of -NH-NH2 group of benzohydrazide in presence of carboxylic group of benzoic acid into 1,3,4-oxadiazole with the objective to develop molecules with excellent yield and less reaction time. Addition of -NH2, -OH group as substituents enhances the yield of the prepared compounds. Among the synthesized compounds, those with electron withdrawing substituents: 5-(4-nitrophenyl)-2-(4-chlorophenyl)-1,3,4-oxadiazole **(XIV) **and 5-(4-nitrophenyl)-2-(4-nitrophenyl)-1,3,4-oxadiazole (**XV) **showed excellent anticonvulsant activity, antidepressant, and antianxiety activity with no neurotoxicity. The result of the present investigation may encourage us to develop and/or improve similar other related compounds and it may be assumed that further modifications may produce compounds of better activity with less side effects.

## Experimental

### Chemical synthesis

All the chemicals and reagents were obtained from Sigma (Germany) and CDH (India) and were recrystallized/redistilled as necessary. Melting points were determined by open capillary tube method and are uncorrected. Purity of the compounds were checked on thin layer chromatography (TLC) plates precoated with silica gel G using solvent system ethyl acetate: petroleum ether (**1) **(3:7 v/v) and benzene: methanol (**2) **(2:1 v/v). The spots were visualized under iodine vapours and UV light. IR spectra were recorded using KBr on a FTIR Shimadzu 8400S IR spectrophotometer (Japan). A JEOL AL300 FTNMR 300 MHz spectrometer was used to acquire ^1^H NMR spectra with DMSO as solvent and tetra methyl silane (TMS) as internal standard. Chemical shift values are expressed in δ ppm. Mass spectra were obtained with a VG70-70H mass spectrometer. Elemental analyses were carried out with a Perkin Elmer model 240-C apparatus. The results of the elemental analysis (C, H, N) are within ± 0.4% of the calculated amounts.

#### General procedure for the synthesis of 2,5-substituted diphenyl-1,3,4-oxadiazoles

**Step 1: **Synthesis of substituted ethyl benzoate (**V-VIII**):

Compounds (**V-VIII**) was synthesized by refluxing a mixture of substituted benzoic acid (I-IV) (0.0587 mol) with absolute ethanol (12 ml) in presence of conc. sulphuric acid (0.5 ml) for 5 h at 40-50°C. Excess of alcohol was distilled off and allowed to cool in ice bath. Reaction was monitored by TLC. The residue thus obtained was separated and washed with water and recrystallized with ethanol [13]. The physico-chemical data of the synthesized compounds (V-VIII) are given in Table 5.

**Table 5:** Physico-chemical data of the synthesized compounds (V-VIII)

Compound	*R*	Molecular formula	Molecular weight	% Yield	m.p. (°C)	Rf^solvent system^
**V**	4-OH	C_9_H_10_O_3_	166.17	56.21	108-110	0.72^a^
**VI**	4-NO2	C_9_H_9_NO_4_	195.17	85.25	114-116	0.78^a^
**VII**	4-NH2	C_9_H_11_NO_2_	165.19	64.28	90-92	0.83^a^
**VIII**	4-Cl	C_9_H_9_ClO_2_	184.62	87.16	127-129	0.76^a^

^a^Ethyl acetate: Petroleum ether (3:7 v/v);

**V: **IR (KBr, cm^-1^): 3581 (O-H stretch), 3040 (Ar-H stretch), 1661 (C = O stretch), 1170 (C-O stretch); ^1^H NMR (300 MHz-DMSO-d6-ppm) δ: 7.78 (d, 2H, Ar-H), 6.84 (d, 2H, Ar-H), 5.2 (s, 1H, OH), 4.19 (m, 2H, CH2), 1.30 (m, 3H, CH3); MS (EI) m/z 166.08 [M^+^].

**VI: **IR (KBr, cm^-1^): 3048 (Ar-H stretch), 1651 (C = O stretch), 1192 (C-O stretch), 1550 (C-NO2 stretch); ^1^H NMR (300 MHz-DMSO-d6-ppm) δ: 8.34 (d, 2H, Ar-H), 8.27 (m, 2H, Ar-H), 4.26 (m, 1H, CH2), 1.32 (m, 1H, CH3); MS (EI) m/z 195.07 [M^+^].

**VII: **IR (KBr, cm^-1^): 3370 (N-H stretch), 3035 (Ar-H stretch), 1643 (C = O stretch), 1214 (C-O stretch); ^1^H NMR (300 MHz-DMSO-d6-ppm) δ: 7.75 (d, 2H, Ar-H), 6.51 (d, 2H, Ar-H), 4.35 (m, 1H, CH2), 3.97 (s, 1H, NH), 1.28 (m, 3H, CH3); MS (EI) m/z 165.03 [M^+^].

**VIII: **IR (KBr, cm^-1^): 30443 (Ar-H stretch), 1668 (C = O stretch), 1243 (C-O stretch), 748 (C-Cl stretch); ^1^H NMR (300 MHz-DMSO-d6-ppm) δ: 7.94 (d, 2H, Ar-H), 7.35 (m, 2H, Ar-H), 4.27 (m, 2H, CH2), 1.26 (m, 2H, CH3); MS (EI) m/z 184.01 [M^+^].

**Step 2: **Synthesis of substituted benzohydrazide (**IX-XII**):

Compounds **(IX-XII) **were synthesized by refluxing a mixture of substituted ethyl benzoate (**V-VIII**) (0.0602 mol) with hydrazine hydrate (5 ml) in absolute ethanol (12 ml) for 8 h at 30-40°C. The reaction mixture was cooled to room temperature and poured in ice with constant stirring. Reaction was monitored by TLC. The precipitate obtained was filtered, washed with water and recrystallized with ethanol to give crystalline product. The physico-chemical data of the synthesized compounds (**IX-XII**) are given in Table 6.

**Table 6:** Physico-chemical data of the synthesized compounds (IX-XII)

Compound	*R*	Molecular formula	Molecular weight	% Yield	m.p. (°C)	Rf^solvent system^
**IX**	4-OH	C_7_H_8_N_2_O_2_	152.1	35.96	132-134	0.74^a^
**X**	4-NO2	C_7_H_7_N_3_O_3_	181.15	58.01	148-150	0.82^a^
**XI**	4-NH2	C_7_H_9_N_3_O	151.17	29.16	142-144	0.75^a^
**XII**	4-Cl	C_7_H_7_ClN_2_O	170.60	53.13	156-158	0.77^a^

^a^Ethyl acetate: Petroleum ether (3:7 v/v);

**IX: **IR (KBr, cm^-1^): 3472 (O-H stretch), 3336 (N-H stretch), 3049 (Ar-H stretch), 1658 (amide C = O stretch); ^1^H NMR (300 MHz-DMSO-d6-ppm) δ: 7.79 (m, 2H, Ar-H), 6.86 (m, 2H, Ar-H), 4.2 (s, 1H, OH), 3.21 (m, 2H, CH2), 2.17 (d, 2H, NH), 1.23 (m, 3H, CH3); MS (EI) m/z 180.01 [M^+^].

**X: **IR (KBr, cm^-1^): 3260 (N-H stretch), 3010 (Ar-H stretch), 1664 (amide C = O stretch), 1511 (C-NO2 stretch); ^1^H NMR (300 MHz-DMSO-d6-ppm) δ: 8.35 (d, 2H, Ar-H), 8.19 (d, 2H, Ar-H), 3.25 (m, 2H, CH2), 1.90 (m, 2H, NH), 1.18 (m, 3H, CH3); MS (EI) m/z 209.05 [M^+^].

**XI: **IR (KBr, cm^-1^): 3368.71 (N-H stretch), 3030.95 (Ar-H stretch), 1642.31 (amide C = O stretch); ^1^H NMR (300 MHz-DMSO-d6-ppm) δ: 7.68 (m, 2H, Ar-H), 6.62 (d, 2H, Ar-H), 3.98 (d, 2H, NH), 3.18 (m, 2H, CH2), 1.81 (d, 2H, NH), 1.25 (m, 3H, CH3); MS (EI) m/z 179.15 [M^+^].

**XII: **IR (KBr, cm^-1^): 3279 (N-H stretch), 3047 (Ar-H), 1672 (amide C = O stretch), 727 (C-Cl stretch); ^1^H NMR (300 MHz-DMSO-d6-ppm) δ: 7.84 (m, 2H, Ar-H), 7.44 (m, 2H, Ar-H), 3.26 (m, 2H, CH2), 2.35 (d, 2H, NH), 1.17 (m, 3H, CH3); MS (EI) m/z 198.63 [M^+^].

**Step 3: **Synthesis of 2,5-substituted diphenyl-1,3,4-oxadiazole (**XIII-XXII**).

An equimolar mixture of substituted benzohydrazide (**IX-XII**) (0.0054 mol) with various substituted benzoic acids (0.0054 mol) was refluxed with phosphorus oxychloride (5 ml) for 2-3 h on water bath at 100°C. Reaction mixture was cooled to room temperature and poured in ice. The precipitate obtained was filtered off, washed with water and further purified by recrystallization with ethanol to give 2,5-substituted diphenyl-1,3,4-oxadiazole. The physico-chemical data of the synthesized compounds (**XIII-XXII**) are given in Table 7.

**Table 7:** Physico-chemical data of the synthesized compounds (XIII-XXV)

Compound	*R*	*R*'	Molecular formula	Molecular weight	% Yield	m.p. (°C)	Rf^solvent system^
**XIII**	4-OH	4-Cl	C_14_H_9_ClN_2_O_2_	272.69	74.11	95-97	0.78^a^
**XIV**	4-NO2	4-Cl	C_14_H_8_ClN_3_O_3_	301.68	82.22	165-167	0.75^a^
**XV**	4-NO2	4-NO2	C_14_H_8_N_4_O_5_	312.24	95.24	127-129	0.67^a^
**XVI**	4-NO2	4-NH2	C_14_H_8_ClN_3_O_3_	301.68	78.59	178-180	0.75^a^
**XVII**	4-OH	4-NH2	C_14_H_11_N_3_O_2_	253.26	65.26	106-108	0.61^b^
**XVIII**	4-NO2	2-Br	C_14_H_8_BrN_3_O_3_	346.14	86.01	120-122	0.82^a^
**XIX**	4-NH2	4-Cl	C_14_H_10_ClN_3_O	271.7	78.06	175-177	0.55^b^
**XX**	4-NO2	4-OH	C_14_H_9_N_3_O_4_	283.24	64.79	104-107	0.66^b^
**XXI**	4-OH	2-Br	C_14_H_9_BrN_2_O_2_	317.14	52.36	102-104	0.74^b^
**XXII**	4-NO2	H	C_14_H_9_N_3_O_3_	267.24	81.79	104-106	0.81^a^

^a^Ethyl acetate: Petroleum ether (3:7 v/v)

^b^Benzene: Methanol (2:1 v/v).

**XIII: **IR (KBr, cm^-1^): 3448 (O-H stretch), 3070 (Ar-H stretch), 2980 (C-H Ar. Stretch), 1593 (C = N stretch of 1,3,4-oxadiazole ring), 1417 (C = C ring stretching), 1275 (C-O-C stretch of 1,3,4-oxadiazole ring), 1016 (C-O stretch of oxadiazole ring), 852 (Ar. C-Cl); ^1^H NMR (300 MHz-DMSO-d6-ppm) δ: 7.39 (d, 2H, Ar-H), 7.31 (m, 2H, Ar-H), 7.28 (m, 2H, Ar-H), 6.85 (d, 2H, Ar-H), 4.4-5.6 (s, 1H, OH); MS (EI) m/z 272.02 [M^+^]; Anal. calcd. for C14H9ClN2O2 (%) C, 59.34; N, 9.52: O, 12.78.

**XIV: **IR (KBr, cm^-1^): 3103 (Ar-H stretch), 2848 (Ar. C-H stretch), 1604 (C = N stretch of 1,3,4-oxadiazole ring), 1527 (C = C ring stretching), 1463 (C-H Bend), 1350 (N-O Assymetric stretch of C-NO2), 1242 (C-O-C stretch of 1,3,4-oxadiazole ring), 1010 (C-O stretch of oxadiazole ring), 865 (C-N stretch of Ar. C-NO2); 838 (Ar. C-Cl); ^1^H NMR (300 MHz-DMSO-d6-ppm) δ: 8.16 (d, 2H, Ar-H), 7.71 (m, 2H, Ar-H), 7.46 (d, 2H, Ar-H), 7.30 (t, 2H, Ar-H); MS (EI) m/z 301.04 [M^+^]; Anal. calcd. for C14H8ClN3O3 (%): C, 55.70; N, 12.73: O, 15.21.

**XV: **IR (KBr, cm^-1^): 3038 (Ar-H stretch), 2941 (C-H Ar. stretch), 1533 (C = N stretch of 1,3,4-oxadiazole ring), 1409 (C = C ring stretching), 1359 (N-O stretch Assymetric N-O stretch of C-NO2), 1278 (C-O-C stretch of 1,3,4-oxadiazole ring), 1074 (C-O stretch of oxadiazole ring); ^1^H NMR (300 MHz-DMSO-d6-ppm) δ: 7.79 (d, 4H, Ar-H), 8.32 (d, 4H, Ar-H); MS (EI) m/z 312.05 [M^+^]; Anal. calcd. for C14H8N4O5 (%) C, 54.21; N, 18.03: O, 25.24.

**XVI: **IR (KBr, cm^-1^): 3440 (O-H stretch), 3406 (N-H stretch), 2952 (C-H Ar. stretch), 1633 (C = N stretch of 1,3,4-oxadiazole ring), 1519 (C = C ring stretching), 1340 (N-O stretch Assymetric N-O stretch of C-NO2), 1249 (C-O-C stretch of 1,3,4-oxadiazole ring), 1072 (C-O stretch of 1,3,4-oxadiazole ring); 854 (C-N stretch of Ar. C-NO2); ^1^H NMR (300 MHz-DMSO-d6-ppm) δ: 8.32 (d, 2H, Ar-H), 7.77 (d, 2H, Ar-H), 7.28 (d, 2H, Ar-H), 6.57 (t, 2H, Ar-H), 3.8-4.2 (d, 2H, NH); MS (EI) m/z 282.03 [M^+^]; Anal. calcd. for C14H10N4O3 (%) C, 61.26; N, 18.87; O, 17.24.

**XVII: **IR (KBr, cm^-1^): 3409 (N-H stretch), 3451 (O-H stretch), 2974 (C-H Ar. stretch), 1625 (C = N stretch of 1,3,4-oxadiazole ring), 1492 (C = C ring stretching), 1263 (C-O-C stretch of 1,3,4-oxadiazole ring), 1012 (C-O stretch of oxadiazole ring), ^1^H NMR (300 MHz-DMSO-d6-ppm) δ: 7.37 (d, 2H, Ar-H), 7.28 (m, 1H, Ar-H), 6.91 (m, 1H, Ar-H), 6.75 (m, 2H, Ar-H), 6.62 (m, 1H, Ar-H), 6.47 (d, 1H, Ar-H), 4.3-5.5 (d, 1H, OH), 3.7-4.9 (d, 2H, NH); MS (EI) m/z 252.62 [M^+^]; Anal calcd. for C14H11N3O2 (%) C, 66.35; N, 17.24: O, 10.35.

**XVIII: **IR (KBr, cm^-1^): 3045 (Ar-H stretch), 1681 (C = N stretch of 1,3,4-oxadiazole ring), 1591 (C = C ring stretching), 1362 (Assymetric N-O stretch of C-NO2), 1247 (C-O-C stretch of 1,3,4-oxadiazole ring), 1087 (C-O stretch of 1,3,4-oxadiazole ring); 854 (C-N stretch of Ar. C-NO2); ^1^H NMR (300 MHz-DMSO-d6-ppm) δ: 8.61 (m, 2H, Ar-H), 7.71 (m, 2H, Ar-H), 7.57 (m, 1H, Ar-H), 7.32 (m, 1H, Ar-H), 7.29 (m, 1H, Ar-H), 7.14 (m, 1H, Ar-H); MS (EI) m/z 344.89 [M^+^]; Anal. calcd. for C14H8BrN3O3 (%) C, 50.27; N, 12.23; O, 13.58.

**XIX: **IR (KBr, cm^-1^): 3448 (N-H stretch), 2924 (C-H Ar. stretch), 1685 (C = N stretch of 1,3,4-oxadiazole ring), 1593.09 (C = C), 1091 (C-O stretch of 1,3,4-oxadiazole ring), 837 (Ar. C-Cl); ^1^H NMR (300 MHz-DMSO-d6-ppm) δ: 7.42 (m, 2H, Ar-H), 7.33 (m, 2H, Ar-H), 7.23 (m, 2H, Ar-H), 6.52 (m, 2H, Ar-H), 3.4-4.7 (m, 2H, NH); MS (EI) m/z 271.09 [M^+^]; Anal. calcd. for C14H10ClN3O (%): C, 62.79; N, 14.37; O, 14.89.

**XX: **IR (KBr, cm^-1^): 3402 (N-H stretch), 3110 (O-H stretch), 2987 (Ar. C-H stretch), 1608 (C = N stretch of 1,3,4-oxadiazole ring), 1525 (C = C ring stretching), 1350 (Assymetric N-O stretch of C-NO2), 1010 (C-O stretch of 1,3,4-oxadiazole ring); ^1^H NMR (300 MHz-DMSO-d6-ppm) δ: 8.25 (m, 2H, Ar-H), 7.74 (m, 2H, Ar-H), 7.31 (m, 2H, Ar-H), 6.79 (m, 2H, Ar-H), 4.5-5.2 (s, 1H, OH); MS (EI) m/z 283.04 [M^+^]; Anal calcd. for C14H9N3O4 (%) C, 60.24; N, 15.43; O, 21.75.

**XXI: **IR (KBr, cm^-1^): 3457 (O-H stretch), 2996 (C-H Ar. stretch), 1632 (C = N stretch of 1,3,4-oxadiazole ring), 1578 (C = C ring stretching), 1213 (C-O-C stretch of 1,3,4-oxadiazole ring), 1075 (C-O stretch of 1,3,4-oxadiazole ring); ^1^H NMR (300 MHz-DMSO-d6-ppm) δ: 7.57 (m, 1H, Ar-H), 7.36 (m, 1H, Ar-H), 7.28 (m, 2H, Ar-H), 7.21 (m, 1H, Ar-H), 7.09 (m, 1H, Ar-H), 6.81 (m, 2H, Ar-H), 4.9-5.4 (s, 1H, OH); MS (EI) m/z 315.98 [M^+^]; Anal. calcd. for C14H9BrN2O2 (%) C, 54.24; N, 8.82; O, 10.94.

**XXII: **IR (KBr, cm^-1^): 3051 (Ar-H stretch), 1618 (C = N stretch of 1,3,4-oxadiazole ring), 1519 (C = C ring stretching), 1282 (C-O-C stretch of 1,3,4-oxadiazole ring), 1059 (C-O stretch of 1,3,4-oxadiazole ring); 849 (C-N stretch of Ar. C-NO2); ^1^H NMR (300 MHz-DMSO-d6-ppm) δ: 8.07 (m, 2H, Ar-H), 7.61 (m, 2H, Ar-H), 7.53 (m, 2H, Ar-H), 7.35 (m, 2H, Ar-H), 7.29 (m, 1H, Ar-H); MS (EI) m/z 267.06 [M^+^]; Anal. calcd. for C14H9N3O3 (%) C, 62.26; N, 15.17; O, 17.74.

### Pharmacology

Albino mice of either sex, weighing (25-30 g) were used for bioactivity studies. The animals were allowed food and water *ad libitum *except during the experiments. They were housed in a room at 25 ± 2°C and 50 ± 5% relative humidity. Animals were obtained from Animal House Facility, Meerut Institute of Engineering and Technology, Meerut. All the test compounds and reference drug were administered orally, suspended in 1% (w/v) carboxymethyl cellulose (CMC) suspension.

### Anticonvulsant activity (MES)

The synthesized compounds were evaluated for their anticonvulsant activity using MES method. The animals were randomly allocated into three groups (standard, control, and test) of six animals each and were fasted for 24 h before the experiment with free access to water. Control group received only 1% (w/v) carboxymethyl cellulose suspension. Standard drug phenytoin was administered orally at a dose of 30 mg kg^-1^. The test compounds were administered orally at an equimolar oral dose relative to 30 mg kg^-1 ^phenytoin. Test compounds and standard drug were administered orally as suspension in carboxymethyl cellulose in water (1% w/v). Supramaximal electroshock of current intensity 50 mA, 60 Hz was given for duration of 0.2 s after the administration of test and standard drug. The anticonvulsant activity was assessed after 30 min of administration. The abolition of hind limb tonic extensor spasm was recorded as a measure of anticonvulsant activity [14]. The results of anticonvulsant activity were summarized in Table 1 and the graphs for anticonvulsant activity were presented in Figures 1 and 2.

### Antidepressant activity (spontaneous motor activity using actophotometer)

The synthesized compounds were evaluated for their antidepressant activity using actophotometer. The animals were randomly allocated into three groups (standard, control, and test) of six animals each and were fasted for 24 h before the experiment with free access to water. Control group received only 1% (w/v) carboxymethyl cellulose suspension. Standard drug fluoxetine was administered orally at a dose of 10 mg kg^-1^. The test compounds were administered orally at an equimolar oral dose relative to 10 mg kg^-1 ^fluoxetine. Test compounds and standard drug were administered orally as suspension in carboxymethyl cellulose in water (1% w/v). In locomotor activity, the basal activity score for all animals and after 60 min of drug treatment for 10 min was recorded [15]. The results of antidepressant activity were summarized in Table 2 and the graph for antidepressant activity was presented in Figure 3.

### Antianxiety activity (elevated plus maze model)

The synthesized compounds were evaluated for their antianxiety activity using actophotometer. The animals were randomly allocated into 3 groups (standard, control, and test) of 6 animals each and were fasted for 24 h before the experiment with free access to water. Control group received only 1% (w/v) carboxymethyl cellulose suspension. Standard drug diazepam was administered orally at a dose of 2 mg kg^-1^. The test compounds were administered orally at an equimolar oral dose relative to 2 mg kg^-1 ^diazepam. Test compounds and standard drug were administered orally as suspension in carboxymethyl cellulose in water (1% w/v). The plus maze apparatus consists of two open arms and two closed arms having an open roof, with the plus maze elevated from the floor was used to observe anxiolytic behavior in animals. Each mouse was placed at the center of the elevated plus maze with its head facing the open arms [16]. During this, the behavior of the mouse was recorded as:

(1) The number of entries into the open arms

(2) Average time spent by the mouse in the open arms

$$\text{Average}\;\text{time}=\frac{\text{Total}\;\text{time}\;\text{spent}\;\text{in}\;\text{open}\;\text{arms}}{\text{Number}\;\text{of}\;\text{entries}\;\text{in}\;\text{arms}}$$

The results of antianxiety activity were summarized in Table 3 and the graphs for antianxiety activity were presented in Figures 4 and 5, respectively.

### Neurotoxicity studies (rotarod animal model)

The disruptive effects on motor coordination were assessed using the Rotarod Tread mill mouse method. The mice were trained to stay on an accelerating rotarod that rotates at 10 revolutions min^-1 ^and 3.2 cm in diameter. Trained mice were given test compounds in dose of 30 mg kg^-1 ^orally. Unimpaired mice can easily remain on a rod rotating at this speed. Neurological deficit, e.g., ataxia, sedation, hyperexcitability is indicated by the inability of the mice to maintain equilibrium on the rod for at least 1 min in each of three concurrent trails [17]. The results of neurotoxic activity measured by rotarod animal model were summarized in Table 6.

### Statistical analysis

Statistical analysis of the anticonvulsant, antidepressant, and antianxiety activity of the synthesized compounds on animals was evaluated using a one-way analysis of variance (ANOVA). In all the cases, post-hoc comparisons of the means of individual groups were performed using Tukey's test. Differences with ***p ≤ 0.001 between experimental groups at each point were considered statistically significant. All values were expressed as mean ± SEM (standard error of mean). For statistical analysis we use sigma stat 2.03 version. (Systat Software, Inc. Point. CA, USA).

## Competing interests

The authors declare that they have no competing interests.
